# Medication errors among elderly in-patients at an urban national referral hospital in Tanzania: Prevalence and patient profiles in a low-resource setting

**DOI:** 10.4314/ahs.v26i1.16

**Published:** 2026-03

**Authors:** Anita Kaligirwa, Emanuel L Peter, Paul E Alele

**Affiliations:** 1 Department of Pharmacology and Therapeutics, Mbarara University of Science and Technology, Mbarara, Uganda; 2 Traditional Medicine Research Centre, National Institute for Medical Research, Mabibo, Dar Es Salaam, Tanzania

**Keywords:** Medication, Errors, Elderly, In-patients, Tanzania

## Abstract

**Background:**

Prescribing processes for the elderly are complex and challenging due to advanced age-related physiologic changes, co-morbidities, and co-medications which increase the chances of medication errors. There are, however, limited studies on the magnitude and profiles of medication errors among elderly populations in low-resource settings such as Tanzania.

**Objective:**

To determine the prevalence and profiles of medication errors among elderly in-patients at Mwananyamala Regional Referral Hospital (MRRH) in Dar-es-Salaam in Tanzania.

**Method:**

Medical data were analyzed of patients aged 65 years and above who were admitted to MRRH between March 2019 and February 2020 for any type of illness. Medication errors were systematically assessed using STOPP/START criteria.

**Results:**

Of 298 patients' records analyzed, the majority were females (n=151, 50.7%). Each patient had at least 2 diseases with an average of 5 types of medicines per patient. Twenty-eight patients (9.40%) had at least one error while the majority (n=270, 90.60%) had no medication errors. Medication omission accounted for the majority of errors present (86, 28.86%).

**Conclusion:**

Medication errors were prevalent among elderly patients admitted at MRRH, with diverse profiles related to the errors. Further studies are warranted to devise and adopt strategies to mitigate medication errors in this population.

## Introduction

With improvements in socio-economic conditions, the population of elderly people has steadily increased worldwide including in developing countries like Tanzania. Globally, the geriatric population is expected to reach 1.5 billion in 2050^1^. This demographic shift calls for countries to improve their health and social systems to ensure effective and safe but cost-effective use of medications and good quality of life. Geriatric prescribing is a complex and challenging process due to age and physiologic-related changes which bring about altered pharmacokinetic and pharmacodynamic effects to the prescribed drugs^2^. Besides, the prescribing is made more complicated by the presence of chronic diseases, nutritional problems, and co-medications^2^.

The American National Coordinating Council for Medical Error Reporting and Prevention (NCC-MERP), defined medication errors as “any preventable event that may cause or lead to inappropriate medication use or patient harm while the medication is in the control of the health care professional, patient, or consumer”^3^. Medication errors consist of prescribing faults or prescription errors. Prescribing faults are attributable to failures in the prescribing process which lead to, or have the potential to lead to, harm to the patient^4^,^5^. Prescription errors are considered as failures in the prescription writing process leading to a wrong instruction regarding one or more of the normal features of a prescription e.g. identity of the drug, formulation, dose, frequency, and duration of administration^4^. We thus defined medication errors as the use of therapeutic drugs that may have led to, had the potential to lead to, or actually harmed the patient^4^. These medication errors included inappropriate prescriptions, irrational prescriptions, underprescription, and overprescription. The prevalence of medication errors remains high across the globe. For example, in Ethiopia, medication errors in the elderly were estimated to be as high as 61.5%^6^ which is close to that reported in the USA at 69%^7^.

Several factors are linked to the high prevalence of medication errors in the elderly, including age and physiological related changes, multiple diseases and medications^8^-^10^. Drug utilization in elderly patients is higher compared to the younger population. In the USA, the elderly constitute 12% of the population but consume 34% of all prescribed drugs^11^. These factors are equally ubiquitous in Tanzania, which could be influencing medication error occurrence.

Due to the complexity of elderly prescribing, screening tools to assess medication errors in prescriptions have been developed, including the STOPP (Screening Tool of Older Persons' Prescriptions)/START (Screening Tool to Alert to Right Treatment) criteria^12^. The STOPP criteria are used by researchers and health care personnel to systematically identify medication errors that are potentially inappropriate medications (PIMs) whereas the START criteria identify potential prescribing omissions (PPOs). Potentially inappropriate medications are those that potentially pose greater risk than benefit to patients^10^. The revised STOPP/START tool consists of a total of 133 PIMs and 57 PPOs^14^. This validated tool has been used in different countries and has yielded comparable results^12^. We used the STOPP/START tool because it has been demonstrated to facilitate medication review in older people with multi-morbid conditions in clinical settings^15^. Specifically, the STOPP/START criteria are designed to detect common potentially inappropriate medications and potential prescribing omissions and thus are suitable for medication review and audit^14^. Also because our study involved a retrospective analysis of the medical records, we deemed that the STOPP/START criteria would give us a better measurement of the medication error profile unlike the Beers Criteria which is limited in recommendations for appropriate dosing, dosing frequency, and medication underuse^13^.

The elderly population requires special attention; however, geriatric health services in Tanzania are poorly developed. Medication errors in the elderly are a common cause of morbidity, mortality, and overutilization of health services. With the increasing elderly population, the quality and safety of medication use have become important aspects of improving their health.

This study, therefore, used a retrospective assessment of treatment records to determine medication errors in patients aged 65 years or above who were admitted to Mwananyamala Regional Referral Hospital (MRRH) in Dar-es-Salaam, Tanzania using the STOPP/START criteria. Because data are scant regarding hospital-based medication errors among elderly in-patients in poorly resourced settings, our study modestly contributes to the data on medication errors generally, and may be used as a baseline in future surveillance studies that would inform policy and practice in developing countries in the region. The findings of this study contribute to a better understanding of the magnitude of medication errors among elderly in-patients in low-resource hospital settings. These findings may thus contribute to formulating evidence-based and rational drug use policies that will help guide appropriate mitigation strategies for medication errors among the elderly in the East African region.

## Materials and methods

### Study design and site

We conducted a cross-sectional study using the medical records of patients admitted at MRRH, located at Kinondoni district in Dar-es-Salaam region, Tanzania. The MRRH is a public health facility serving a population of more than 2.2 million in Dar-es-Salaam and the surrounding areas. It has a bed capacity of 254.

### Study population and sample size

The study population consisted of medical records of all patients aged 65 years and above, admitted in the medical ward of MRRH between March 2019 and Feb 2020. For sample size calculation, we considered prevalence of medication errors of 25.5% as reported in Fadare et al., 2013 in Nigeria, since the Nigerian population is likely to share similar characteristics to the Tanzanian one^2^. Morever, a margin of error of 5% and 95% confidence gave a total of 291, hence the study included 300 records of eligible patients.

### Inclusion and exclusion criteria

All medical records of patients aged 65 years and above who were admitted to the medical ward at MRRH between March 2019 and Feb 2020 were included. On the other hand, all records with incomplete information about patients' demographics, medications used at the hospital, and those without date of admission were excluded.

### Sampling technique and sample selection

A simple random sampling technique was adopted in which the sampling frame was a list of all patients admitted at the medical ward during the study window. Then, we randomly selected 300 records using random numbers generated by Microsoft Office Excel 2010. Before data extraction, the investigator guided by the medical officer assessed the completeness of the medical records according to pre-determined exclusion criteria.

### Data management and analysis

Study numbers were used as unique identifiers in the data capturing form. Data were cleaned and checked for completeness before entry into EpiData version 3.1 database. Later, data were exported to STATA (version 14.0 StataCorp LP) software for analysis. The first stage of analysis included data summarization. For continuous variables, mean, median, standard deviation, and range were calculated while categorical variables were summarized into proportions and presented in a tabular form. The second stage of analysis involved testing the associations of several patients' and clinical variables with the occurrence of medication errors. A Chi-square test, t-test, and Fisher's exact test were applied to measure associations. A p-value <0.05 was considered statistically significant.

## Ethical considerations

The study protocol received ethical clearance (number 05/06-20) from the Mbarara University of Science and Technology Research Ethics Committee (MUST-REC) and ethical clearance (number NIMR/HQ/R.8a/Vol.IX/3773) from Medical Research Coordinating Committee (MRCC) of the National Institute for Medical Research, Tanzania. Data from the medical records were anonymized using study numbers. Personal identifiers such as patients' names were not extracted. All medical records were treated as confidential documents. Permission to access data was obtained from the medical officer in charge (MOI) of MRRH.

## Results

### Demographic characteristics

This study included 298 elderly patients with a mean age of 73.4 ±7.9 years. Females were the majority, representing 50.7% of the participants. Distribution of age according to groups showed that the majority of the patients were between the age group of 65-74 years accounting for 67.1%, followed by those who were between 75-84 years (19.1%) while those aged 85 and above were 13.8% (Table 1).

**Table 1:** Characteristics of elderly patients admitted at MRRH medical ward

Characteristics	N (%)
**Age in years**	
65-74	200(67.11%)
75-84	57(19.12%)
85>	41(13.76%)
**Mean age (73.4), SD (7.3)**	
**Sex**	
Female	151(50.67%)
Male	147(49.33%)
**Duration of hospitalization (days)**	
<8	281(94.30%)
8-15	15(5.03%)
>15	2(0.67%)
**Discharge outcome**	
Normal	216(72.48%)
Death	46(15.44%)
Referred to MNH	31(10.40%)
Taken home due to deterioration	5(1.68%)

Hospitalization profile: About 94.3% of the patients stayed in the hospital for less than 8 days, followed by those that stayed between 8 to 15 days, accounting for 5.0%. Out of the 298 patients, 216 (72.5%) were discharged normally, 31 (10.4%) patients were referred to Muhimbili National Hospital, 5 (1.7%) patients asked to go home due to deterioration, while 46 (15.4%) patients were reported dead (Table 1).

**Figure 1:**
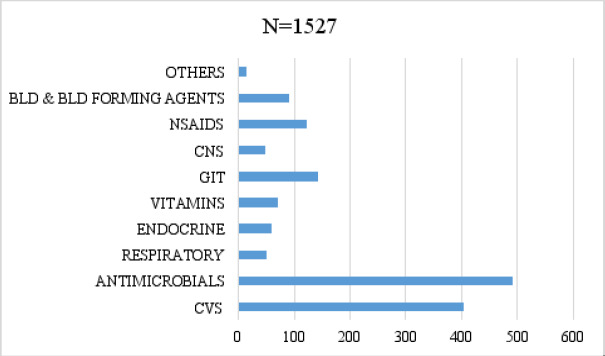
Profile of prescribed medicines

A total of 1523 drugs were prescribed. Antimicrobial drugs were the most prescribed drugs representing 32.2%, followed by drugs acting on the cardiovascular system (CVS) with 26.5%, drugs acting on the gastro-intestinal tract (GIT) with 9.3%, and the NSAIDS representing 8.% of the total (Fig 1).

**Figure 2:**
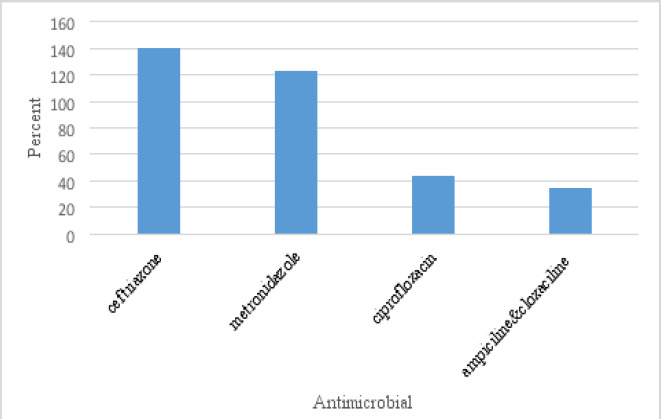
Top four highly prescribed antimicrobial medicines

Among the antimicrobials, the most prescribed drug was ceftriaxone with 28.6%, followed by metronidazole with 25.1%, ciprofloxacin 8.9% and ampicillin-cloxacillin combination 8.9% (Fig. 2).

**Figure 3:**
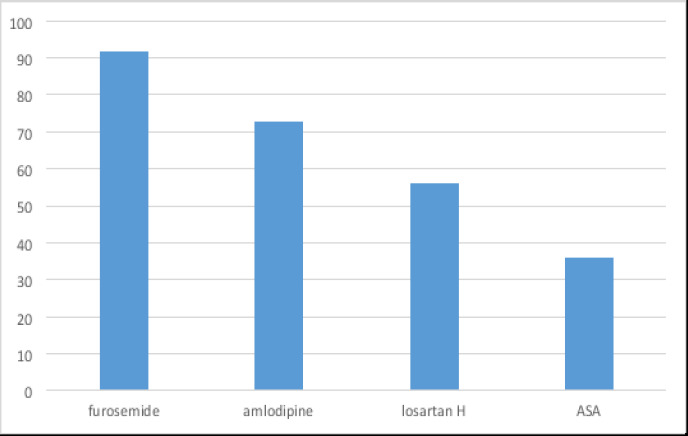
Top four highly prescribed medicines acting on the cardiovascular system

Regarding drugs acting on the cardiovascular system, furosemide was the most prescribed (22.8%). Followed by amlodipine (18.1%), Losartan H (13.9%), while aspirin 75 mg (aspirin junior) accounted for 8.9% (Fig 3).

**Figure 4:**
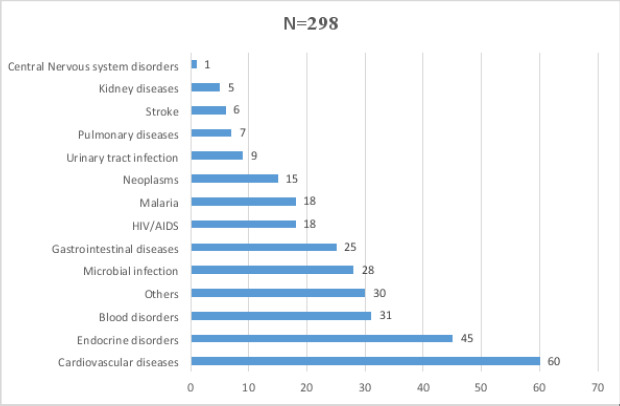
Profile of diseases among elderly patients

The commonest cause of morbidity was cardiovascular disease accounting for 20.10%, followed by the endocrine disorders representing 15.1%, blood disorders representing 10.4%, and microbial infections representing 9.4% of the total morbidity, (Fig 4).

### Prevalence of medication errors

A total of 28 (9.40%) patients had at least one medication error as per STOPP criteria. Regarding number of errors per patient, 27 patients had one medication error while only one had 2 medication errors. The most common medication error was the use of sulphonylureas with a long duration of action in elderly patients with type 2 diabetes, which is associated with increased risk of prolonged hypoglycemia. The other error involved long term use of aspirin at doses greater than 160 mg per day which increases the risk of bleeding to the patients. Three cases involved prescribing drugs without any evidence-based clinical indication. One patient was prescribed metformin while having hypoglycemia which may have exacerbated the hypoglycemia. Similarly, two patients with hypotension were prescribed furosemide which could have exacerbated hypotension (Table 2).

**Table 2:** Prevalence of medication errors

Medication error	Contraindicated risk	Observed medication error (N=29)
Any drug prescribed without an evidence-based clinical indication		3(10.34%)
Long-term aspirin at doses greater than 160mg per day	Increased risk of bleeding, no evidence for increased efficacy	4(13.79%)
NSAID with severe hypertension or severe heart failure	Risk of exacerbation of hypertension or risk of exacerbation of heart failure	2(6.90%)
Sulphonylureas with a long duration of action (e.g. glibenclamide, chlorpropamide, glimepiride) with type 2 diabetes mellitus	Risk of prolonged hypoglycemia	19(65.52%)
Aspirin, clopidogrel, dipyridamole, vitamin K antagonists, direct thrombin inhibitors or factor Xa inhibitors with concurrent significant bleeding risk, i.e. uncontrolled severe hypertension, bleeding diathesis, recent non-trivial spontaneous bleeding	High risk of bleeding	1(3.45%)
**Total**		29(100%)

Medication errors may increase morbidity and mortality in elderly patients. This may increase duration of the hospitalization thus increasing treatment costs to the patient. However, our study did not find a significant association between medication error and duration of hospitalization (p >0.05). The majority of patients with medication errors stayed for less than 8 days in the hospital (Table 3).

**Table 3:** Association between medication error and duration of hospitalization

Medication error as per STOPP	Duration of hospitalization (days)			
	<8	8-15	>15	Total
**No**	259(86.91%)	9(3.02%)	2(0.67%)	270(90.60%)
**Yes**	27(9.06%)	1(0.34%)	0(0.00%)	28(9.40%)
**Total**	286(95.97%)	10(3.36%)	2(0.67%)	298(100%)
	**P=0.899**			

There was no significant association between medication errors and the discharge outcome (p > 0.05). The majority of patients who had medication errors also had normal hospital discharge (Table 4).

**Table 4:** Association between medication error and discharge outcome

Medication error	Admission outcome					
	Asked to go home	Death	Normal	Referred to MNH	Taken home due to deterioration	Total
**No**	1(0.34%)	42(14.09%)	195(65.44%)	28(9.40%)	4(13.42%)	270(90.60%)
**Yes**	0(0.00%)	4(13.42%)	21(7.05%)	3(1.00%)	0(0.00%)	28(9.40%)
**Total**	1(0.34%)	46(15.44%)	216(72.48)	31(10.40%)	4(13.42%)	298(100%)
	**P = 0.966**					

### Omitted Medications (OM)

A total of 86 (28.86%) patients had medications omitted, where 30 (34.88%) had at least 1 medication omitted, 50 (58.14%) had 2 medications omitted and 6 (6.98%) had 3 medications omitted. This makes a total of 149 medications omitted to the patients. “Statin therapy with a documented history of coronary, cerebral or peripheral vascular disease, unless the patient's status is end-of-life or age is > 85 years”, was the most medication omitted accounting for 48.32%. Another major medication omitted was “Antiplatelet therapy (aspirin or clopidogrel or prasugrel or ticagrelor) with a documented history of coronary, cerebral or peripheral vascular disease” accounting for 37.58% (Table 5).

**Table 5:** Proportion of medication omissions

Drugs	Omitted medication N(%)
Angiotensin converting enzyme inhibitor (ACEI) with systolic heart failure and/or documented coronary artery disease	1(0.67%)
Antihypertensive therapy where systolic blood pressure consistently > 160 mmHg and/or diastolic blood pressure consistently > 90 mmHg; if systolic blood pressure > 140 mmHg and/or diastolic blood pressure > 90 mmHg, if diabetic	1(0.67%)
Antiplatelet therapy (aspirin or clopidogrel or prasugrel or ticagrelor) with a documented history of coronary, cerebral or peripheral vascular disease	56(37.58%)
Aspirin therapy in patients with well controlled BP	17(11.41%)
Beta blocker with ischemic heart disease	1(0.67%)
Statin therapy with a documented history of coronary, cerebral or peripheral vascular disease, unless the patient's status is end-of-life or age is > 85 years	72(48.32%)
Xanthine-oxidase inhibitors (e.g. allopurinol, febuxostat) with a history of recurrent episodes of gout	1(0.67%)
**total**	**149(100%)**

Omitted medications to the patients may cause increase in the duration of the hospitalization which in turn increases the costs of treatment to the patient. In this study, 96.7% of the patients with potentially omitted medication (POM) stayed in the hospital for less than 8 days. Therefore, the study did not show a significant association between the hospital stay and the POM (Table 6).

**Table 6:** Association between omitted medication and duration of hospitalization

POM as per START	Duration of hospitalization (days)			
	<8	8-15	>15	Total
**No**	202(67.79%)	8(2.68%)	2(0.67%)	212(71.14%)
**Yes**	84(28.19%)	2(0.67%)	0(0.00%)	86(28.86%)
**Total**	286(95.97%)	10(33.56%)	2(0.67%)	298(100%)
	**P=0.541**			

Potentially omitted medications may increase morbidity and comorbidity in the elderly patients. However, 64% of the patients who had POM, also had a normal hospital discharge. Our study did not establish a significant relationship between POM and discharge outcome (Table 7).

**Table 7:** Association between potentially omitted medications (POM) and discharge outcome

POM as per START	Admission outcome					
	Asked to go home	Death	Normal	Referred to MNH	Taken home due to deterioration	Total
**No**	1 (0.30%)	32 (10.74%)	153 (51.34%)	23 (7.72%)	3 (1.01%)	212 (71.14%)
**Yes**	0 (0.00%)	13 (4.36%)	64 (21.48%)	8 (2.68%)	1 (0.30%)	86 (28.86%)
**Total**	1 (0.30%)	45 (15.01%)	217 (72.82%)	31 (10.40%)	4 (1.34%)	298 (100%)
	**P = 0.959**					

## Discussion

The goal of this study was to determine the prevalence and profiles of medication errors among elderly in-patients at Mwananyamala Regional Referral Hospital (MRRH) in Dar-es-Salaam in Tanzania. The medication errors in this study included inappropriate prescriptions, irrational prescriptions, underprescription, and overprescription. We evaluated medication errors using STOPP/START criteria in elderly patients admitted in the medical ward at MRRH. We chose these criteria because of the retrospective nature of our study that did not involve recruitment of study participants. Studies have shown that applying STOPP/START criteria has a number of benefits including reducing adverse drug reactions, improving medication appropriateness, reducing polypharmacy, and lowering medication costs^15^. Use of these criteria have also been shown to improve clinical outcomes in multimorbid older people^15^. Our study contributes to the body of evidence regarding vulnerable elderly patients living in low-resource settings who experience medication-related errors that can adversely affect their treatment course.

We found that the presence of medication errors at MRRH was quite significant among the elderly in-patients admitted at MRRH. To the best of our knowledge, this study is among the few studies conducted in Tanzania and the region using the START/STOPP criteria. The prevalence of medication errors was found to be 9.4% using the criteria in the STOPP tool. Our results were comparable to a study conducted in Turkey whose prevalence was 9.8%16, but lower than a study conducted in Nigeria whose prevalence was 25.5%^2^. However, our study prevalence was higher than in a study conducted in Croatia whose prevalence was 2.2%. The low proportion of patients with medication errors in the Croatian study could be due to the use of computerized systems in the pharmacy to detect the medication errors, and intensive training to their health care workers^17^.

The most common medication error accounting for 62.3% in this study was the use of sulphonylureas with a long duration of action (e.g. glibenclamide, chlorpropamide, glimepiride) with type 2 diabetes mellitus, which is associated with prolonged hypoglycemia. In a meta-analysis performed in 2007, glibenclamide was associated with hypoglycaemia more frequently when compared with other sulfonylureas, and it is a significant medical concern in the type II diabetic geriatric population. Sulfonylureas act on the pancreatic beta cell channels (ATP-K channel) to facilitate insulin secretion thus reducing diabetes-associated hyperglycemia^18^. Compared to glipizide or glimepiride, glibenclamide has a higher affinity for pancreatic beta-cell sulfonylurea receptors, greater tendency for accumulation of active metabolites, and greater penetration of pancreatic tissue^18^,^19^. As a result, glibenclamide has a prolonged half-life of about 10 hours compared to an average half-life of 5 hours for glimepiride and 2 to 5 hours for glipizide^20^,^21^.

The elimination and volume of distribution of glibenclamide are also increased to a greater extent for glibenclamide compared to glimepiride and glipizide^25^,^26^. Usually sulfonylureas are hepatically and renally cleared, but in the elderly they are slowly eliminated due to the age-associated decrease in renal function^25^,^26^. Thus a combination of the above factors can lead to increased insulin release for longer periods after cessation of the medication, especially in decreased renal function, as can be the case in the elderly^21^. The 18th WHO Expert Committee on the Selection and Use of Essential Medicines (18th EC) compiled and analyzed comparative evidence for safety and efficacy of second generation sulphonylureas with a focus on glibenclamide, glimepiride, glipizide, and gliclazide and concluded that glibenclamide is not a safe medication for use in the elderly (patients older than 60 years of age^24^.

“Statin therapy with a documented history of coronary, cerebral or peripheral vascular disease, unless the patient's status is end-of-life or age is > 85 years” was the most omitted medication error in our study accounting for 72%. This was followed by “Antiplatelet therapy (aspirin or clopidogrel or prasugrel or ticagrelor) with a documented history of coronary, cerebral or peripheral vascular disease” accounting for 56%. Aspirin and statin therapy have important roles in cardiovascular diseases by reducing the incidence of cardiovascular accidents and mortality in elderly patients. In a meta-analysis that included 14,483 participants older than 75 years, statin treatment was associated with a 15% reduced rate of major cardiovascular events (OR 0.85, 95% CI 0.73–0.98) among those with existing atherosclerotic cardiovascular disease^25^. Another meta-analysis of statin trials in 2016 revealed that each 1 mmol/L reduction in LDL cholesterol decreases the yearly rate of major atherosclerosis cardiovascular events by one fifth and total mortality by 10%^26^. In conclusion, aspirin and statins therapy should not be neglected in elderly patients given their huge importance in the well-being of the patients.

The most common cause of morbidity was cardiovascular disease accounting for 20.1%, followed by endocrine disorders at 15.1%, blood disorders at 10.4% and microbial infections at 9.4%. This corresponds to the systemic analysis for the global burden of disease in 2019, which reported that the most notable common cause of morbidity was cardiovascular disease in the elderly patients of 70 years and older^27^. According to this study, the second prevalent disease in our set-up was endocrine disorders of which 98% of these disorders was diabetes mellitus type 2. This corresponds to the study by Fadare et al. in which diabetes was also the second most prevalent in that setting with the prevalence of 6.4%^2^. Decline in lean body mass and increase in body fat, particularly visceral adiposity that often accompanies aging, may contribute to the development of insulin resistance. As for the mechanism of type 2 diabetes mellitus, it is known that aging induces a decrease of insulin sensitivity and insufficient compensation of beta cell functional mass in the face of increasing insulin resistance^28^. Related to beta cell function, aging correlates with a decrease of beta cell proliferation capacity and increases sensitivity to apoptosis^29^.

Despite the cardiovascular and the endocrine disorders being the most prevalent, the majority of medicines prescribed were the antimicrobials accounting for 32.1% of the total drugs prescribed. Among the reasons for high antimicrobial prescriptions is difficulty in determining whether an elderly patient truly needs an antimicrobial agent. This is due to lack of typical signs and symptoms of infections in older adults which narrows the fine line between colonization and infection, leading to a higher suspicion of infections, thus increased use of antimicrobials^30^.

Because this was a retrospective analysis of medical data, our study had some limitations. First, this study could not determine the source of the medication errors for the categories involving prescription faults and errors. It is possible that the medication errors encompassed knowledge-based errors in prescribing such as occurred for the prescriptions involving long-acting sulfonylureas in the elderly patients; or the errors could also have been technical errors such as in the prescription for high dose Aspirin. It is also possible that some other sources of errors were involved in the medication errors seen in this study. Regardless, the prescription faults identified could lead to, or had the potential to lead to, harm to the patients^4^. Second, the retrospective analysis could not document the actual harm from the medication errors and distinguish actual harm to the patients from potentially inappropriate medications. The medical records used in the analysis did not document suspected harms as a result of the medications prescribed. Thus, this retrospective analysis while assessing the potentially inappropriate medications does not necessarily indicate findings that actual harm occurred to those patients. Nevertheless, this study contributes data on the prevalence of medication errors among geriatric patients living in low-resource settings. There are scant data on this vulnerable population in Africa, despite the growing numbers of elderly patients in these settings. Coupled with the few numbers of medical personnel trained in geriatrics and the inadequacies in geriatric healthcare services, these data contribute to the evidence needed to design interventions aimed at reducing morbitity and mortality arising from medical (prescription) errors and fostering improvements in the care of these patients.

## Conclusion

Based on the study findings, we conclude that the prevalence of medication errors among elderly patients attending regional referral hospitals in Tanzania remain high. The medication errors arose from prescribing faults that included inappropriate prescriptions, irrational prescriptions, underprescription, and overprescription. There is therefore need to plan for interventional studies to devise and adopt strategies to mitigate medication errors and ultimately improve the quality of healthcare among the elderly.
